# Identification of quantitative trait loci of pod dehiscence in a collection of soybean grown in the southeast of Kazakhstan

**DOI:** 10.18699/vjgb-24-58

**Published:** 2024-09

**Authors:** B.N. Doszhanova, A.K. Zatybekov, S.V. Didorenko, T. Suzuki, Y. Yamashita, Y. Turuspekov

**Affiliations:** Institute of Plant Biology and Biotechnology, Almaty, Kazakhstan Al-Farabi Kazakh National University, Almaty, Kazakhstan; Institute of Plant Biology and Biotechnology, Almaty, Kazakhstan; Kazakh Research Institute of Agriculture and Plant Growing, Almalybak, Almaty region, Kazakhstan; Hokkaido Research Organization, Sapporo, Japan; Hokkaido Research Organization, Sapporo, Japan; Institute of Plant Biology and Biotechnology, Almaty, Kazakhstan Al-Farabi Kazakh National University, Almaty, Kazakhstan

**Keywords:** soybean, pod dehiscence, seed yield, genome-wide association study, quantitative trait locus, соя, растрескивание бобов, урожай зерна, полногеномный анализ, локусы количественных признаков, QTL

## Abstract

Soybean [Glycine max (L.) Merr.] is one of the important crops that are constantly increasing their cultivation area in Kazakhstan. It is particularly significant in the southeastern regions of the country, which are currently predominant areas for cultivating this crop. One negative trait reducing yield in these dry areas is pod dehiscence (PD). Therefore, it is essential to understand the genetic control of PD to breed new cultivars with high yield potential. In this study, we evaluated 273 soybean accessions from different regions of the world for PD resistance in the conditions of southeastern regions of Kazakhstan in 2019 and 2021. The field data for PD suggested that 12 accessions were susceptible to PD in both studied years, and 32 accessions, in one of the two studied years. The genotyping of the collection using a DNA marker for the Pdh1 gene, a major gene for PD, revealed that 244 accessions had the homozygous R (resistant) allele, 14 had the homozygous S (susceptible) allele, and 15 accessions showed heterozygosity. To identify additional quantitative trait loci (QTLs), we applied an association mapping study using a 6K SNP Illumina iSelect array. The results suggested that in addition to major QTL on chromosome 16, linked to the physical location of Pdh1, two minor QTLs were identified on chromosomes 10 and 13. Both minor QTLs for PD were associated with calmodulin-binding protein, which presumably plays an important role in regulating PD in dry areas. Thus, the current study provided additional insight into PD regulation in soybean. The identified QTLs for PD can be efficiently employed in breeding for high-yield soybean cultivars.

## Abstract (ru)

Соя [Glycine max (L.) Merr.] – одна из важнейших сельскохозяйственных культур, площади которой в Казахстане постоянно увеличиваются. Особенно эта культура значима в южных и юго-восточных регионах страны, которые являются основными регионами выращивания сои. К негативным факторам, влияющим на урожайность сои в засушливых районах, относится растрескивание стручков. Поэтому понимание генетического механизма растрескиваемости стручков сои важно для выведения новых высокоурожайных сортов. В настоящем исследовании мы изучили 273 сорта и линии сои из разных регионов мира на устойчивость к растрескиваемости в условиях Южного Казахстана в 2019 и 2021 гг. Наблюдения за признаком «растрескиваемость стручков сои» в полевых условиях Алматинской области выявили, что в 2019 г. растрескиванию были подвержены 23 сорта, в 2021 г. – 21 сорт. Двенадцать сортов сои повторно подвергались растрескиванию в оба года эксперимента. Согласно средним данным испытаний, всего подвержены растрескиванию 32 сорта сои. При генотипировании коллекции с использованием ДНК-маркера гена Pdh1, основного гена растрескиваемости стручков сои, у 244 образцов был выявлен устойчивый аллель, у 14 образцов – восприимчивый, а 15 образцов обладали гетерозиготностью. Для идентификации дополнительных локусов количественных признаков (quantitative trait locus, QTL) мы применили полногеномный анализ с использованием 6 тысяч SNP-маркеров на основе чипа 6K SNP Illumina iSelect. В дополнение к основному QTL на хромосоме 16, связанному с физическим расположением гена Pdh1, были идентифицированы два минорных QTL на хромосомах 10 и 13. Оба минорных локуса ассоциированы с растрескиванием стручков сои и связаны с кальмодулин-связывающим белком, который, вероятно, играет важную роль в регулировании растрескиваемости стручков сои в засушливых регионах. Таким образом, нами получена дополнительная информация о регуляции растрескиваемости в сое. Идентифицированные QTL для признака «растрескиваемость стручков сои» могут быть эффективно использованы при селекции высокоурожайных сортов сои.

## Introduction

Soybean [Glycine max (L.) Merr.] is a major crop among oilseeds worldwide and a global source of edible protein and oil, providing approximately 60 and 28 % of the world supply, respectively (Vollmann et al., 2000; Zhou et al., 2020). According to the USDA, Brazil, the United States of America, and Argentina are the largest soybean production countries, while Kazakhstan is on the list of the top forty producers (https://ipad.fas.usda.gov). Kazakhstan is one of the largest agro-industrial countries in Central Asia and is interested in increasing soybean production areas (Abugalieva et al., 2016; Didorenko et al., 2016; Zatybekov et al., 2017). Therefore, developing new competitive cultivars for new cultivation areas is a priority for the local breeding community.

One of the limiting factors for the increase in soybean productivity, particularly in southern regions, is pod dehiscence (PD), which leads to a substantial yield loss (Zhang Q. et al., 2018). For wild plants, PD is an important mechanism for spreading progenies (Benvenuti, 2007; Fuller, 2007), but for cultivated plants, it is an unfavorable agronomic trait because mature pods open to release seeds before harvesting (Kang et al., 2009; Zhang L., Boahen, 2010). PD was nearly eliminated during soybean domestication and breeding (Liu et al., 2007; Krisnawati, Adie, 2017). Nevertheless, the yield losses due to PD today may range from 34 to 99 % depending on genetic background, environmental factors, pod morphology and anatomy, and management (Romkaew, Umezaki, 2006; Bhor et al., 2014; Parker et al., 2021).

Pod dehiscence is a highly heritable and complex trait; it was shown that its broad sense heritability may range from 90 to 98 % in different populations (Tsuchiya, 1987; Bailey et al., 1997; Kang et al., 2009). Previously, two genes, Pdh1 and SHAT1-5, were identified and mapped on chromosome 16 (Funatsuki et al., 2008, 2014; Dong et al., 2014). The gene pdh1 was identified in cultivated soybeans by Funatsuki and co-authors in 2014 (Funatsuki et al., 2014). The dominant Pdh1 encodes a dirigent family protein in soybean and is highly expressed in the pod endocarp layer, increasing dehiscing forces. The recessive pdh1 in dehiscence-resistant types includes a premature stop codon, which blocks proper protein synthesis (Funatsuki et al., 2014). The effect of pdh1 on pod dehiscence is generally larger among the other genes that had important value in worldwide soybean cultivation (Funatsuki et al., 2014; Hu et al., 2019; Zhang J., Singh, 2020). SHAT1-5 gene activates secondary wall synthesis and stimulates the dehiscence site’s thickening in pods. The domestication process resulted in extra SHAT1-5 expression compared to the wild soybean allele (Dong et al., 2014). Previous research suggested that all domesticated soybeans carry SHAT1-5 haplotypes derived from a haplotype that differs from wild soybeans (Funatsuki et al., 2014; Sedivy et al., 2017).

Recently, a genome-wide association study (GWAS) described another dehiscence-associated candidate gene, Glyma09g06290 (Hu et al., 2019). This gene is highly expressed in developing pods; however, the biological functions of this gene should be further investigated (Hu et al., 2019). Later, another GWAS showed that the NST1A gene (Glyma.07G050600) has a potential role in soybean pod dehiscence (Zhang J., Singh, 2020). NST1A codes a NAC family transcription factor and a paralog of SHAT1-5 (NAC are NAM, ATAF1/2, and CUC2 proteins, the largest families of transcription factors in plants: NAM – no apical meristem proteins, ATAF1/2 – Arabidopsis transcription activation factor, CUC2 – cup-shaped cotyledon; NST1-NAC secondary thickening1) (Zhang J., Singh, 2020). The authors identified an indel in its coding sequence, leading to a premature stop codon. Epistatic analyses showed that NST1A works with Pdh1 to provide durable resistance to pod dehiscence (Zhang J., Singh, 2020; Parker et al., 2021).

Apart from genes, several QTLs were repeatedly identified throughout the soybean genome on different chromosomes. To date, several QTLs for PD have been identified on almost all chromosomes in different soybean populations (Bailey et al., 1997; Liu et al., 2007; Kang et al., 2009; Yamada et al., 2009; Han et al., 2019; Hu et al., 2019). The identified QTL on chromosome 16 was located near the major gene pdh1 and had a high value of the coefficient of determination (Seo et al., 2020; Jia et al., 2022).

Most new QTLs were identified using GWAS, a powerful tool for detecting natural variation involving the regulation of complex traits based on genotype-phenotype association (Rafalski, 2010; Huang, Han, 2014). Although many QTLs for PD in soybeans were discovered, some can be unstable in different environments and may vary in diverse genetic backgrounds (Hu et al., 2019; Seo et al., 2020; Jia et al., 2022). Hence, additional studies for searching QTLs for PD are important for breeding practices in new soybean environments. Therefore, this study aimed to identify QTLs for PD in the southeast region of Kazakhstan using a diverse world soybean collection.

## Materials and methods

Field evaluation of the collection. The soybean collection consisted of 273 cultivars and lines from Eastern and Western European countries, North America, and East and Central Asia (Supplementary Material 1)1 (Zatybekov et al., 2017, 2018).The collection was grown in 2019 and 2021 at the experimental stations of Kazakh Research Institute of Agriculture and Plant Growing (KRIAPG, Almaty region, Kazakhstan) located at an altitude of 740 m above sea level, 43°15′ N, 76°54′ W (Doszhanova et al., 2019). This site is characterized by continental climatic conditions: mild and cool winters, cool spring, hot and dry summers, and warm and dry fall. The meteorological data registered for the experiments are provided in Supplementary Material 2. The collection was planted in four rows per plot, 25 cm plant spacing, 50 cm row spacing, and 1 m row length without soil fertilizers. 


Supplementary Materials are available in the online version of the paper: https://vavilovj-icg.ru/download/pict-2024-28/appx19.pdf


The yield component traits screened in soybean accessions are the number of fruiting nodes (NFN, pcs), the number of seeds per plant (NSP, pcs), yield per plant (YP, g), thousand seed weight (TSW, g). The PD data was collected by visually estimating the percentage of pods at the R8 stage in a plot that had dehisced at the full maturity stage on a scale of 1–5, where 1 ≤ 1–20 %, 2 ≤ 21–40 %, 3 ≤ 41–60 %, 4 ≤ 61–80 % and 5 ≤ 81–100 % (Supplementary Material 1). Correlation analysis was conducted using RStudio software (Allaire, 2011).

DNA extraction and PCR procedure. DNA was extracted from young leaves by a modified CTAB method (Suzuki et al., 2012). Amplification of DNA was performed using an allele-specific PCR method with four primers for the SNP marker of the Pdh1 gene associated with pod dehiscence in soybean (Funatsuki et al., 2014). PCR reaction of 10 μl of the solution containing the DNA template (50 ng/μl), AmpliTaqGold MasterMix (Applied Biosystems by Thermo Fisher Scientific), two pairs of primers (forward and reverse outer primers, forward and reverse inner primers), and M13 primer, labeled with fluorescent (FAM, NED, VIC and PET, Applied Biosystems). PCR amplification used an initial 95 °C for 7 min; 35 cycles of 94 °C for 30 sec, 56 °C for 30 sec, and 72 °C for 1 min, and a final 72 °C extension for 7 min. PCR products were analyzed on an ABI Prism 3500 Genetic Analyzer (Applied Biosystems) with GeneMapper software as described previously (Suzuki et al., 2012).

Linkage disequilibrium, population structure, and genome-wide association study. For GWAS, the genomic DNA of all samples in the collection was genotyped using the 6K SNP Illumina iSelect array (Song et al., 2013) at the Trait Genetics Company (TraitGenetics GmbH Gatersleben, Germany). SNP genotype analysis was carried out using Illumina Genome Studio software (GS V2011.1). The quality control of genotyped data was performed by filtering SNPs with call rate ≥90 % and minor allele frequency (MAF) ≥5 %. Accessions with missing data being greater than 10 % were removed. SNP loci with more than 10 % heterozygous calls were also removed (Bradbury et al., 2007). Pairwise linkage disequilibrium (LD) between the markers based on their correlations (R2) was calculated using TASSEL. R statistical software was used to plot the correlation between pairwise R2 and the genetic distance, LD decay plot (www.R-project.org).

The population structure (Q) analysis was performed using STRUCTURE software version 2.3.4 (Pritchard et al., 2000). The optimal number of clusters (K) was chosen based on the ΔK as described by (Evanno et al., 2005). The obtained values were then transformed into a population structure (Q) matrix. The kinship matrix (K) was generated by TASSEL software V5.0 (Bradbury et al., 2007).

GWAS was conducted based on the Mixed Linear Model (Q + K) using TASSEL software V5.0 (Bradbury et al., 2007). The statistical significance thresholds, Bonferroni correction, and alternative method False Discovery Rate (FDR) were used to distinguish true positives from false positives and false negatives. The significance level of 5 % after Bonferroni multiple test correction was used to identify significant associations (Buckler et al., 2011). The Benjamini–Hochberg procedure was calculated to control the FDR threshold at 5 % (Benjamini, Hochberg, 1995). The SoyBase database (www. soybase.org) was used to search genes for identified markertrait associations.

## Results

Field experiments and traits evaluation

**Fig. 1.**
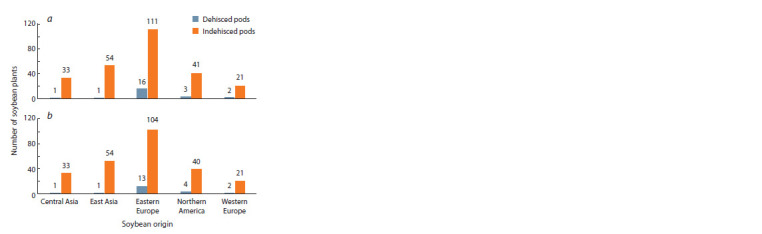
The field screening of the world soybean collection by the pod dehiscence trait in 2019 (a) and 2021 (b) years of experiments.

Observing PD in the field conditions of the Almaty region showed that 23 accessions in 2019 and 21 accessions in 2021 dehisced their pods in the field conditions (Fig. 1), and 12 accessions repeatedly fully or almost fully dehisced their pods with grade 4 or 5 in two years of experiments in the Almaty region (Supplementary Material 1).

The results of two years of experiments showed that the vast majority of the soybean collection was resistant to PD in the Almaty region conditions, but 32 accessions were found to be susceptible to PD in one of the two years of study. After harvesting, the soybean collection was analyzed by yield components, such as NSP, NFN, YP, and TSW. The soybean collection studied in the Almaty region was more productive in 2021 than in 2019. The average values of two years for NFN, NSP, YP, and TSW were 15.01 nodes, 37.88 seeds, 9.62 g, and 149.12 g, respectively. The ranges of soybean yield components in the Almaty region in two experimental years and average data are shown in Table 1

**Table 1.**
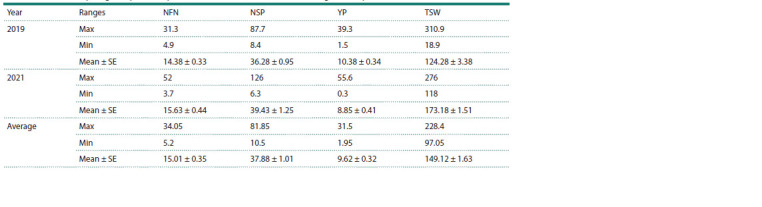
The variability ranges of yield component traits in 2019, 2021, and the average of two years Note. NFN – number of fruiting nodes (pcs), NSP – number of seeds per plant (pcs), YP – yield per plant (g), TSW – thousand seed weight (g), SE – standard error.

**Fig. 2.**
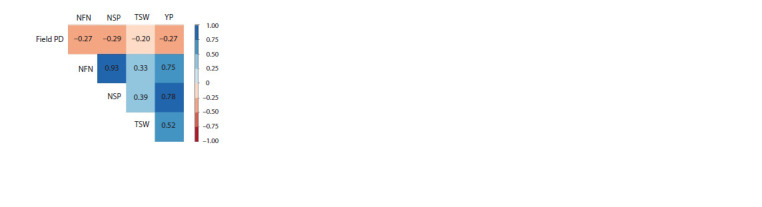
Correlation analysis of the pod dehiscence trait in the field conditions and yield components Field PD – pod dehiscence in the field conditions

Pearson correlation analysis suggested that the average data of the PD trait in the field conditions of the Almaty region were negatively and significantly associated with all yield components, NFN, NSP, YP, and TSW, with coefficients of correlation –0.27, –0.29, –0.2, and –0.27 respectively ( p < 0.01, RStudio). In their turn, NSP, YP, and TSW had a significant and positive correlation with each other ( p < 0.01) (Fig. 2). 

Genotyping of soybean collection

**Fig. 3.**
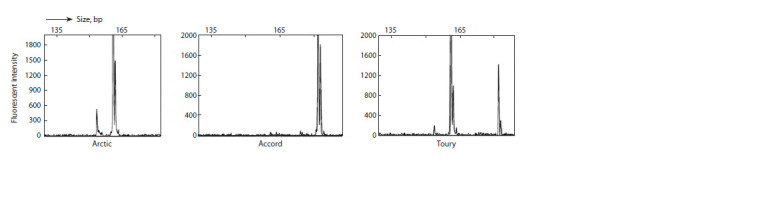
Amplification products of specific SNP marker for the Pdh1 gene in Arctic, Accord, and Toury soybean varieties with S and R alleles and heterozygote (H), respectively, detected by Genetic Analyzer 3500.

The soybean collection consisted of 273 samples and was genotyped using four primers for the SNP marker of the Pdh1 gene, which is associated with PD. The SNP analysis of soybean accessions identified three alleles: S – pod dehiscence susceptible, R – pod dehiscence resistant, and H – heterozygous (Fig. 3). A t-test with significance confirmed the difference among groups of three alleles at p < 0.001.

**Fig. 4.**
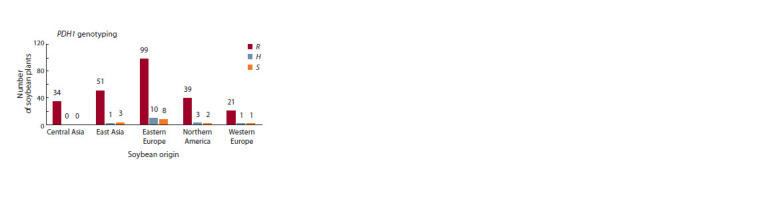
The genotyping results of the soybean collection studied using an allele-specific SNP marker of the Pdh1 gene. S – homozygous genotypes with the susceptible allele, R – homozygous genotypes with the resistant allele, H – heterozygotes.

The results of Pdh1 genotyping using an allele-specific SNP marker showed that 244 out of 273 accessions were with the homozygous R (resistant) allele, 14 had the homozygous S (susceptible) allele, and 15 samples were heterozygotes (Supplementary Material 1). Figure 4 illustrates the distribution of alleles of different origins in the soybean collection.

Most of the accessions carrying the susceptible S alleles in homozygous or heterozygous genotypes were from Eastern Europe (10 and 8 accessions, respectively). In accessions from East Asia, three cultivars were with the homozygote S allele (ʻKheikhek14ʼ, ʻDong doe 641ʼ and ʻKen feng 20ʼ, China), and one was heterozygous (ʻKharbinʼ, China). In accessions from Northern America, two cultivars were with the homozygous S allele (ʻKG 20ʼ, Canada and ʻCarolaʼ, USA), and three were heterozygous genotypes (ʻMaple Arrowʼ and ʻGEOʼ, Canada and ʻLinkolnʼ, USA). In accessions from Western Europe, one cultivar carried the S allele (ʻSepiaʼ, France), and one was heterozygous (ʻFiskeby5ʼ, Sweden). All Central Asian accessions carried the homozygous R allele of the pdh1 (Fig. 4).

The results of field screening for PD of the average data for the two years of experiments and genotyping data by DNA marker showed a moderate correlation link ( p < 0.01). Comparative assessment of PD in field studies and Pdh1 genotyping indicated that in 14 accessions with the homozygous S allele, only seven cultivars were susceptible to PD in both years, and ten samples, in one of the two studies years (Supplementary Material 1). These seven cultivars were from Eastern Europe (6 accessions) and Northern America (1 accession). In 244 identified samples with the homozygous R allele, four accessions were susceptible to PD in both years, and 19 accessions, in at least one out of two studied years (Supplementary Material 1). These four cultivars were from Eastern Europe (3) and North America (1).

Linkage disequilibrium, population structure, and genome-wide association study.

**Fig. 5.**
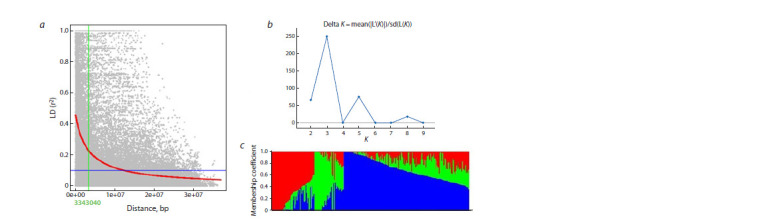
a, LD decay plot of 4,651 SNPs through the whole soybean genome; b, Delta K for differing numbers of subpopulations; c, bar plot of estimated population structure of 273 soybean genotypes on K = 3.

After filtering the genotyping data by MAFs, missing data in individuals, and heterozygous calls, a total of 4,651 SNPs remained. The average density of the SNP map was one marker per 246 Kb. Linkage disequilibrium (LD) decayed at 3.3 Mb for the whole genome at R2 of 0.1 (Fig. 5a). The population structure (Q) based on the results of STRUCTURE and STRUCTURE Harvester analyses showed three subpopulations (Fig. 5c). The Q matrix was developed using K = 3 as the optimum (Fig. 5b.

**Fig. 6.**
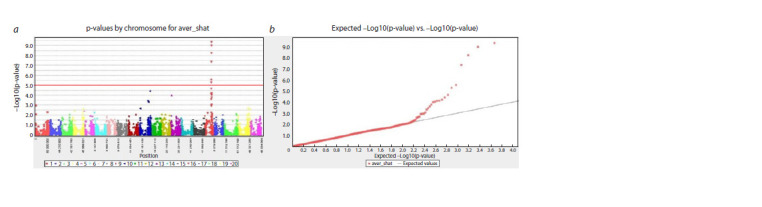
Manhattan (a) and QQ plots (b) for the pod dehiscence trait in the world soybean collection for average data of 2019 and 2021 in the Almaty region.

The Manhattan plot with SNP markers associated with PD and the QQ plot are illustrated in Figure 6, the Manhattan plot and the QQ plot of each year of the experiment are illustrated in Supplementary Materials 3, 4. The threshold is 1.0×10–5 at a significance level of 5 % after Bonferroni multiple test correction. A significance threshold of 5 % FDR was used to identify putative SNP associations. If two SNPs were closer than the genome average LD decay value of 3.3 Mbp, they were considered to belong to the same locus.

The GWAS with significance thresholds of FDR and Bonferroni correction allowed the identification of three QTLs for PD on chromosomes 10, 13, and 16 (Fig. 6, Table 2, Supplementary Materials 3–5). For each identified QTL, one most significant SNP marker with the lowest p-value was selected: Gm10_47774781 on chromosome 10, Gm13_6207590 on chromosome 13, and Gm16_29681065 on chromosome 16. The information about the marker positions on the chromosomes, p-values, effects, and phenotypic variations for alleles is shown in Table 2.

**Table 2.**
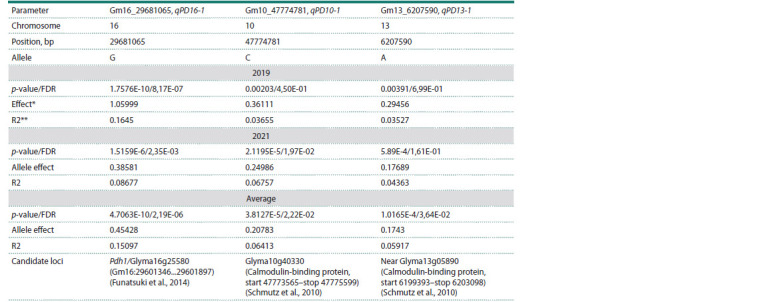
The list of identified significant SNP markers associated with PD for 2019 and 2021 and the average data for the two years of the experiment using the genome-wide association study * Absolute effect; ** R2 – marker phenotypic variation.

Gm16_29681065 was located in the vicinity of Pdh1 on chromosome 16 (Table 2). Other two minor QTLs were identified on chromosomes 10 and 13. Identified SNPs with the most significant p-values of Gm16_29681065, Gm10_47774781, and Gm13_6207590 were designated as qPD16-1, qPD10-1, and qPD13-1.

The influence of the allelic status of the most significant SNPs of three stable QTLs for the PD phenotype is shown in Table 3. The results in Table 3 indicate that the combination of effective SNP alleles (TTG) in three QTLs resulted in PD resistance with a value of 0.1. In contrast, the combination of alternative alleles (GCA) showed susceptibility to PD with a value of 3.9. Interestingly, two plants with the TCA combination (a resistant allele for Gm16_29681065 and two susceptible alleles for Gm10_47774781 and Gm13_6207590) showed PD phenotype with the value of 4.5 (Table 3), suggesting that the effective allele in Gm16_29681065 alone is not sufficient for PD resistance.

**Table 3.**
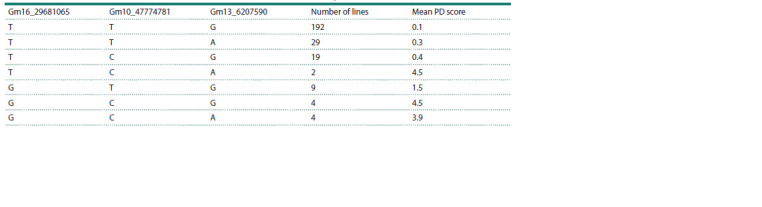
Mean of PD scores for allelic combinations of SNP markers in three identified quantitative trait loci of PD in field conditions

## Discussion

The assessment of the collection in the field conditions of the southeast of Kazakhstan has confirmed a high negative impact of PD on yield performance (Fig. 2). The field evaluation of average data revealed that 32 genotypes were susceptible to PD in at least one of the two studied years (Fig. 1). The phenotypic results for PD over two years of study were stable and largely coincided with genotypic results using an allele-specific SNP marker of Pdh1, confirming the fact that Pdh1 played a critical role in soybean expansion (Funatsuki et al., 2014). Nevertheless, 19 out of 244 accessions with homozygous R alleles showed susceptibility to PD in the field conditions of southeast Kazakhstan, suggesting that more genes are involved in regulating PD. Therefore, GWAS was applied to identify additional genetic factors that can potentially be involved in the genetic control of PD. The application of GWAS suggested that three stable QTLs for PD were significant in this study

The three identified QTLs (qPD10-1, qPD13-1, and qPD16- 1) were located on chromosomes 10, 13, and 16, respectively (Table 2). As QTL qPD16-1 was highly significant both in 2019 and 2021, it can be considered a major genetic factor showing a remarkable effect on PD. The location of QTN qPD16-1 coincided with the genetic position of Pdh1 (Funatsuki et al., 2014) (Table 2). The literature survey suggests that Pdh1 (Gm16:29601346–Gm16:29601897) encodes a dirigent family protein known to be involved in lignification, which increases dehiscing forces by promoting torsion of dried pod walls (Funatsuki et al., 2014). The loss-of-function pdh1 gene has been widely used in soybean breeding as a pod dehiscence resistance gene (Funatsuki et al., 2014).

The other significant SNP for PD identified on chromosome 10, qPD10-1, was located in Glyma10g40330 (Schmutz et al., 2010), the gene that is responsible for the expression of plant calmodulin-binding protein (soybase.org). Previously, another QTL for PD was identified on chromosome 10, which was located within 10 cM of Satt243 (Gm10:46088332– 46088382, soybase.org) (Kang et al., 2009), suggesting a strong genetic linkage between QTNs in two association findings. Interestingly, the significant QTL identified on chromosome 13 was located in the vicinity of Glyma13g05890, which is also expressing plant calmodulin-binding protein (Schmutz et al., 2010; soybase.org).

The results of influences of all three identified genetic factors on PD performance suggest that although the role of qPD16- 1 is remarkable, the allelic statuses of Gm10_47774781 and Gm13_6207590 are also essential (Table 3). Hence, it can be hypothesized that calmodulin-binding protein is part of the gene network controlling PD. Calmodulin (CAM) is a Ca2+ sensor known to regulate the activity of many eucaryote proteins and plays an important role in plant growth and development (Yu et al., 2021). An increasing number of studies have illustrated that plant calcium signals play a vital role in life processes by acting as a messenger transducer in the complicated signal network to regulate plant growth and development and the response and adaptation to environmental stresses (Hong-Bo et al., 2008). Hypothetically, drought or high temperature as environmental stress can induce responses by activating calmodulin-binding protein, leading to a change in the structure of soybean pods. In general, the results of the soybean PD study in conditions of southeast Kazakhstan suggest that it is controlled by one major and two minor QTLs, which is congruent with results of previous reports, where one major and few minor QTLs were revealed (Tsuchiya, 1987; Bailey et al., 1997; Ogutcen et al., 2018; Seo et al., 2020). Nevertheless, qPD13-1, identified in this work, has not been reported in any previous PD studies, and, therefore, it can be considered a putatively novel genetic factor for the regulation of PD in soybeans.

## Conclusion

The evaluation of the collection consisting of 273 soybean accessions with different origins for PD has confirmed a strong influence of the Pdh1 gene on trait performance and a negative impact on yield and yield components over two studied seasons in southeast Kazakhstan. The application of GWAS has allowed the identification of one major (qPD16-1) and two minor (qPD10-1 and qPD13-1) QTLs for PD. The location of the major QTL has coincided with the physical position of the Pdh1. Two minor QTLs have been associated with the genes for calmodulin-binding protein on chromosomes 10 and 13. The assessment of available scientific reports for the genetic control of PD suggests that the QTL for PD on chromosome 13 is a novel genetic factor for regulating the studied trait.

## Conflict of interest

The authors declare no conflict of interest.
